# Deletion of Mitochondrial Translocator Protein (TSPO) Gene Decreases Oxidative Retinal Pigment Epithelial Cell Death via Modulation of TRPM2 Channel

**DOI:** 10.3390/biology10050382

**Published:** 2021-04-28

**Authors:** Dilek Özkaya, Xinhua Shu, Mustafa Nazıroğlu

**Affiliations:** 1 Department of Ophthalmology, Faculty of Medicine, Suleyman Demirel University, Isparta TR-32260, Turkey; drdilekozkaya@yahoo.com; 2 Department of Biological and Biomedical Sciences, Glasgow Caledonian University, Glasgow G4 0BA, UK; xinhua.shu@gcu.ac.uk; 3 Department of Vision Science, Glasgow Caledonian University, Glasgow G4 0BA, UK; 4 School of Basic Medical Sciences, Shaoyang University, Shaoyang 422000, China; 5 Neuroscience Research Center, Suleyman Demirel University, Isparta TR-32260, Turkey; 6 Drug Discovery Unit, BSN Health, Analyses, Innovation, Consultancy, Organization, Agriculture, Ltd., Isparta TR-32260, Turkey

**Keywords:** ARPE19 cells, cell death, mitochondrial translocator protein, mitochondrial oxidative cytotoxicity, TRPM2 channel

## Abstract

**Simple Summary:**

18 kDa mitochondrial translocator protein (TSPO) is a mitochondria protein of the cellular outer membrane in the mitochondria of several cells, including ARPE19 is TSPO. Accumulating evince indicates that the presence of TSPO participated the modulations of Ca^2+^ homeostasis and mitochondrial free reactive oxygen species (fROS) generation. The deletion of TSPO gene provides to study the action of TSPO on the levels of apoptosis, ADP-ribose (ADPR), mitochondria-fROS (Mito-fROS), and apoptosis via the stimulation of Ca^2+^ permeable channels in the models of cell culture. The stimulations of oxidative stress and ADPR induce the activation of TRPM2 in the ARPE19. For clarifying the involvement of TSPO in retinal human diseases, we used the ARPE19 human cell culture model. The current results demonstrated that the deletion of TSPO induces the regulation of TRPM2 in the TSPO gene knockout ARPE19 (ARPE19-KO) In fact, the present results show that the presence of TSPO increased the upregulations of apoptosis and mitochondria oxidative cytotoxicity values via stimulation of TRPM2 in the ARPE19. Nevertheless, the blockages of PARP-1 (PJ34 and DPQ) and TRPM2 (2APB and ACA) downregulated the values of cell death and oxidative cytotoxicity in the ARPE19. In summary, present results clearly demonstrate that the deletion of TSPO decreases mitochondrial oxidative cytotoxicity-mediated cell death via the modulation of TRPM2 in the ARPE19.

**Abstract:**

The current results indicated the possible protective actions of 18 kDa mitochondrial translocator protein (TSPO) deletion on TRPM2 stimulation, mitochondrial free ROS (Mito-fROS) and apoptotic harmful actions in the cells of adult retinal pigment epithelial19 (ARPE19). There was a direct relationship between TSPO and the disease of age-related macular degeneration. The nature of TSPO implicates upregulation of Mito-fROS and apoptosis via the activation of Ca^2+^ channels in ARPE19, although deletion of TSPO gene downregulates the activation. The decrease of oxidative cytotoxicity and apoptosis might induce in TSPO gene deleted cells by the inhibition of Mito-fROS and PARP-1 activation-induced TRPM2 cation channel activation. The ARPE19 cells were divided into two main groups as TSPO expressing (ARPE19) and non-expressing cells (ARPE19-KO). The levels of caspase -3 (Casp -3), caspase -9 (Casp -9), apoptosis, Mito-fROS, TRPM2 current and intracellular free Ca^2+^ were upregulated in the ARPE19 by the stimulations of H_2_O_2_ and ADP-ribose, although their levels were downregulated in the cells by the modulators of PARP-1 (DPQ and PJ34), TRPM2 (ACA and 2APB) and glutathione. However, the H_2_O_2_ and ADP-ribose-mediated increases were not observed in the ARPE19-KO. The expression levels of Bax, Casp -3, Casp -9 and PARP-1 were higher in the ARPE19 group as compared to the ARPE19-KO group. In summary, current results confirmed that TRPM2-mediated cell death and oxidative cytotoxicity in the ARPE19 cells were occurred by the presence of TSPO. The deletion of TSPO may be considered as a therapeutic way to TRPM2 activation-mediated retinal oxidative injury.

## 1. Introduction

Age-related macular degeneration (AMD) disease causes blindness in old people [1]. The incidences of AMD with eleven million patients are high in the USA [2]. The incidence of AMD in Turkish patients is also high (15.3%) [3]. Drusen are the accumulation of yellow deposits in the subretinal pigment epithelial and they are associated with the etiology of AMD [4,5]. The drusen contain high concentration of the lipids [6]. The unsaturated fatty acid contents in the lipids are the main targets of free reactive oxygen species (fROS) in several cells, including retinal cells [7,8,9]. The dry AMD isn’t totally treated by the current drugs. However, accumulation data indicate that inhibition of excessive fROS generation in the membranes of retinal mitochondria is a reasonable way for the treatment of dry AMD disease [1,10,11].

The outer membrane of mitochondria allows the influx of calcium ion (Ca^2+^) [10]. The high expression level of 18 kDa mitochondrial translocator protein (TSPO) was indicated in the outer membrane of mitochondria [10]. TSPO has a function on the translocation of cholesterol into the mitochondrial matrix, and its name is coming from function [12]. In a previous study, a high amount of TSPO expression levels in the cells of adult retinal pigment epithelial-19 (ARPE19) was reported [13] and it participated in the modulation of Ca^2+^ influx from the primary microglia cells of human [12]. The involvement of TSPO was shown in the regulation of various cellular and mitochondrial functions [12,14]. Accumulating evidence indicates that TSPO has a multifunctional nature on the arrangements of mitochondrial oxidative phosphorylation, the production of fROS, inflammation and overload Ca^2+^ influx in several cells [12,15]. Moreover, the modulator action of TSPO was also reported on the functions of cell viability and apoptosis in the retina cells [14,16,17].

In mammalian, the superfamily of transient receptor potential (TRP) cation channel has 28 members within 7 subgroups [18,19]. A member of the TRP superfamily is TRPM2 cation channels and it is activated in the several cells such as ARPE19 and dorsal rood ganglion (DRGs) by several stimuli including the fROS, nitric oxide, NADPH-oxidase and ADP-ribose (ADPR) [20,21,22]. In ARPE19 cells, the activation of TRPM2 is responsible from the upregulation of intracellular free Ca^2+^ concentration ([Ca^2+^]_c_), apoptosis, mitochondrial membrane depolarization (mMDP) and fROS [21,23]. There is also a direct relationship between the generation of mitochondrial fROS and the activation of TRPM2 in the ARPE19. Similarly, accumulating data indicate that there is a direct relationship between the presence of TSPO activity and the activity of voltage-dependent cation channel (VDCC) for the regulation of [Ca^2+^]_c_ [14]. The TSPO gene deletion-mediated decrease on the expression levels of VDCC in the human microglia was recently reported [12]. The deletion of TSPO modulated [Ca^2+^]_c_ and oxidative cell injury by decreasing [Ca^2+^]_c_ via the modulation of VDCC [14]. In addition, the TSPO induced apoptotic action via the activation of NADPH-oxidase in the microglia and eye cells of mice by producing fROS, although the action was not observed in the microglia and eye cells of TSPO knock out mice [24,25]. Hence, TSPO may induce the upregulation of mitochondrial fROS, apoptosis, and [Ca^2+^]_c_ via activation of TRPM2 channel in ARPE19 cells, although the upregulation of apoptotic and oxidant values may be modulated by the deletion of TSPO. 

Our research, therefore, concentrated on investigating the possible protective actions of the TSPO gene deletion in the TRPM2-mediated harmful effects such as apoptosis and oxidative toxicity in the ARPE19. The ARPE19 cell was originated by the differentiation of the human retinal pigment epithelium. Hence, the cell has similar physiologic and morphologic properties of human retinal pigment epithelium such as tight junctions and Bruch’s membranes [26]. The cells of ARPE19 were used as a model of h-RPE in the previous observations [21,23,27]. For the reason, we preferred the cells in the present study.

## 2. Material and Methods

### 2.1. Cell Culture

The ARPE19 and ARPE19 TSPO knock-out (ARPE19-KO) cells were obtained from a co-author (Dr. Xinhua Shu). There were limited reports on the naturel presence of TRPM2 protein in the ARPE19 [20,21], although the naturel presence of TRPM2 in the SH-SY5Y neuroblastoma cell was confirmed by the several observations [28,29,30,31]. For the reason, we further confirmed the naturel presence of TRPM2 via the analyses of Western blot in the ARPE19 by using the positive control cells (SH-SY5Y). The cell culture medium mixture (45%/45%) was prepared by using DMEM-LPX and HAM-12-A (Capricorn Scientific GmbH, Ebsdorfergrund, Germany). The medium mixture was additionally contained FBS (10%) and antibiotics (1%) nutrient medium mixture as described in a previous observation [32]. The T25 and T75 cell culture flasks with filter caps (INTERLAB, Istanbul, Turkey) and 35-mm dishes with bottom glasses (Mattek Corporation, Istanbul, Turkey) were incubated in a NB-203QS incubator (Bucheon, Gyeonggi-do, South Korea). The cells in the T75 flasks and 35-mm dish were used for the Western blot/antioxidant (spectrophotometer) analyses and laser confocal microscope, respectively, although cells in the T25 flask were used for the analyses of automatic plate reader (Infinite 200 PRO, Tecan Life Sci Austria GmbH, Groedig, Austria) and patch-clamp analyses. 

### 2.2. Experimental Design

After inducing two main groups (ARPE19 and ARPE19-KO), the cells in the both groups were divided into three subgroups as follows

*Control group*: The control cells were cultured in the incubator (5% CO_2_ and 37 °C) without the treatment of 2-aminoethoxydiphenyl borate (2APB), H_2_O_2_ and N-(p-amylcinnamoyl) anthranilic acid (ACA) for 1 h. 

*Stimulation group*: The cells in the group were stimulated by extracellular H_2_O_2_ (1 mM) for 5 min or intracellular ADPR [20,21,30,31].

*Treatment group*: The cells in treatment (H_2_O_2_ + 2APB or H_2_O_2_ + ACA) groups were incubated with ACA (25 µM) or 2APB (100 µM) for 5 min after or before the H_2_O_2_ stimulation. The ACA and 2APB were dissolved in the DMSO before the incubation in the extracellular buffer with Ca^2+^ [20,21,30,31].

### 2.3. Obtaining TSPO Knockout (ARPE19-KO) Cells

The details of TSPO gene preparations in the ARPE19 cell were indicated in a previous study [33]. Briefly, the procedure of clustered regularly interspaced short palindromic repeats (CRISPR) was applied for inducing ARPE19-KO cells. After designing the CRISP primers, the Oligos of CRISP were ligated into linearized gRNA vector by generating a CRISP TSPO-gRNA. The TSPO knockout colonies were confirmed by using the analyses of Western blotting and immunocytochemistry (Data were shown in the [33]).

### 2.4. The Determination of Intracellular Free Ca^2+^ Concentration ([Ca^2+^]_c_) 

We assayed TSPO deletion-induced changes of [Ca^2+^]_c_ via imaging the florescence intensity of Fluo-3AM (1 µM for 60 min) (#F-1242, Thermo Fisher Scientific GmbH, Dreieich, Germany) in the ARPE19 previously described [30,31]. For the stimulation of the Fluo-3AM in the cells, argon laser at 488 nm in the LSM 800 confocal microscope setup (Zeiss, Ankara, Turkey) with 40 × 1.3 oil objective was used. Before the H_2_O_2_ stimulation, Fluo-3AM from the cells was removed by washing with extracellular buffer. The fluorescence intensity changes in the cells were analyzed in a computer by using the ZEN program (Zeiss). Arbitrary unit (a.u.) was used for the expression of the Fluo-3 results. 

### 2.5. Patch-Clamp Electrophysiology Analyses

The USB 10 amplifier and Patch-master software were purchased from the HEKA GmbH (Lamprecht, Germany), and it was used in the current study for recording the patch-clamp electrophysiology currents. Before recording the currents, the patch pipettes of the borosilicated capillary tubes were prepared in the puller (P-97 model, Sutter Inc., USA). The ADPR (1 mM) was solved in the intracellular buffer, and the pipettes were filled with the intracellular buffer. After clamping the holding potential at −60 mV, the whole cell configuration of TRPM2 was obtained in the USB 10 by using the patch pipette with high intracellular [Ca^2+^]_c_ [34]. The contents of standard intracellular and extracellular buffers were shown in previous studies [18,23]. The TRPM2 antagonist (ACA and 25 µM in the patch chamber) was used for blocking the TRPM2 currents. The unit of pA/pF was used for the expression of the current density result.

### 2.6. The Assay of Cell Viability and Apoptosis 

The cells (1 × 10^6^) were incubated in the 96-well black plates. After washing the cells with 1xPBS, the MTT (200 µL) from stock solution (0.5 mg/mL) was added to each black well. The plates were kept for 3–4 h in the NB-203QS incubator. The optic density changes of MTT were recorded at 490 nm in the infinite 200 PRO. The protein contents of the cells were manually measured by using Bradford’s reagent. 

The concentration of apoptosis in the cells were spectrophotometrically (Cary 60 UV-Vis, Agilent Inc., Izmir, Turkey) assayed at 550 nm by using an APOPercentage commercial kit (Biocolor Ltd., Northern Ireland). After calculating the results as optic density/mg protein, the concentrations of apoptosis and MTT are shown as % of control.

### 2.7. The Assays of Caspase -3 (Casp -3) and -9 (Casp -3)

The Ac-DEVD-AMC and *Ac*-LEHD-AFC are two fluorogenic substrates of passive Casp -3 and Casp -9 and they can be specifically cleaved by the active Casp -3 and Casp -9. The caspase substrates were purchased form Bachem AG (Bubendorf, Switzerland). The activities of Casp -3 and Casp -9 were quantified in the Infinite 200 PRO by using the fluorescent detection of free AMC and AFC. The AMC was excited at 360 nm and emitted at 460 nm, although the AFC was excited at 400 nm and emitted at 505 nm [30,31]. After normalization to total protein concentration, the fluorescence levels of free AMC and AFC were expressed as % of control.

### 2.8. The Analyses of Mitochondrial fROS Generations 

The non-fluorescent version of MitoTracker Red (#M-7512, ThermoFisher, Istanbul, Turkey) stain is MitoTracker Red CM-H_2_XRos (Mito-fROS) and it is converted to fluorescent form under upon oxidation and laser stimulation. The fluorescent dye stains fROS in the mitochondria of live cells. Before the analyses of Mito-fROS, the cells were incubated with MitoTracker Red CM-H2Xros (1 µM for 30 min). After washing the dye with 1× PBS, the generation of fROS in the mitochondria was imaged in the LSM 800 confocal microscope [30,31]. The fluorescence intensities of MitoTracker Red CM-H2Xros were measured in 15 mm^2^ of each cell for calibration of the intensities. The images were evaluated in a computer by using the ZEN program. The results (n = 25–30) were expressed as a.u.

### 2.9. The Assay of the Mitochondrial Membrane Depolarization (mMDP) 

For the assay of mMDP, the cells in the cell culture dishes were stained with 5 µM JC-1 (Cayman Inc., Istanbul, Turkey) and they were kept in the incubator at dark for 30 min [30,31]. The JC-1 was excited at 595 nm in the LSM 800, although it was emitted at 535 nm. The fluorescence intensities of JC-1 were measured in 15 mm^2^ of each cell for calibration of the intensities. The images were evaluated in a computer by using the ZEN program. The results of mMDP (n = 25–30) were expressed as a.u.

### 2.10. The Analyses of Western Blot 

The analyses of Western blot (n = 3) were performed by using the standard Western blotting methods. After washing the ARPE19 and ARPE19-KO with ice cold 1× PBS, the protein concentration was measured by using the Bradford’s solution. The details of Western blot procedure were indicated in the Refs [30,31]. The primary and secondary antibodies of human TRPM2, PARP-1, Bax, Bcl-2, Casp -3 and Casp -9 were purchased form Cell Signaling Technology (EKA Biolab Teknoloji, Ankara, Turkey). The Gel Imagination System (G:Box, Syngene Inc., Cambridge, UK) was used for imaging the bands. The β-actin antibody was used as an internal control. The band intensities of Bax, Bcl-2, Casp -3, Casp -9, TRPM2 and PARP-1 in the ARPE19-KO were normalized relative to ARPE19 values. 

### 2.11. The Analysis of Reduced Glutathione (rGSH), Glutathione Peroxidase (GSHPx) and Lipid Peroxidation (Malondialdehyde, MDA) 

The levels of rGSH and GSHPx in the ARPE19 and ARPE19-KO cells were analyzed at 412 nm by using a spectrophotometer (Cary 60 UV-Vis, Agilent), although the level of MDA was analyzed at 532 nm as described in the previous studies [23,30]. The data of rGSH and MDA were expressed as μg/g protein in the cells, although the activity of GSHPx was expressed as IU/g protein.

### 2.12. Statistical Analysis

The current data were expressed as mean ± standard deviation (SD). First, we checked whether the presence of statistical significance in the current data by one-way ANOVA test of the SPSS program. Then, the *p* (≤0.05) values of the individual significances were analyzed by using the Student’s *t*-test. 

## 3. Results

### 3.1. The Presence of Nature TRPM2 in the ARPE19 Cells Was Confirmed by Using the Positive Control Cells (SH-SY5Y)

**Figure 1:**
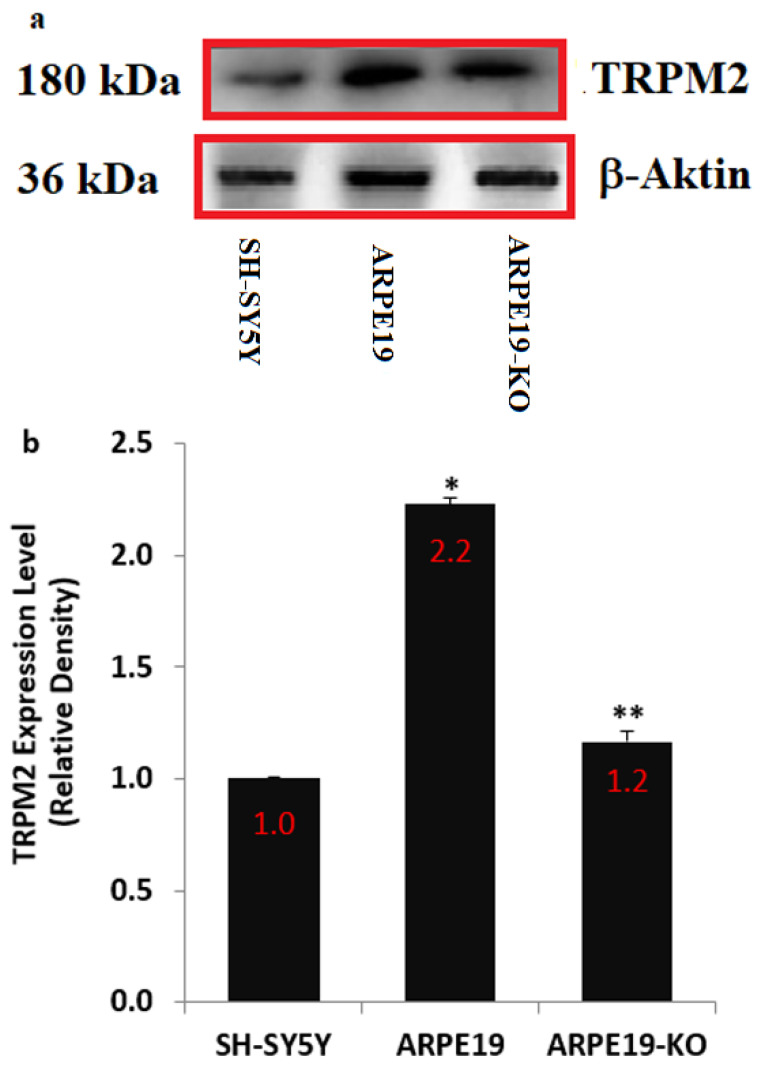
**The expression level of TRPM2 protein**. (Mean ± SD and n = 3). For the expression levels of TRPM2 protein in the cells of SH-SY5Y, ARPE19 and ARPE19-KO, we used standard Western blot analyses. The protein bands of β-actin were used as control. (**a**) The protein bands of β-actin and TRPM2. (**b**) The mean levels of the band proteins in the column figure were expressed as Mean ± SD. 1:500. (* *p* ≤ 0.05 vs. SH-SY5Y cells. ** *p* ≤ 0.05 vs. ARPE19 cells).

There are two reports on the presence of nature TRPM2 in the ARPE19 [20,21], although the presence of nature TRPM2 in the SH-SY5Y cells was reported by the results of several studies [28,29,30,31]. In addition, the TRPM2 channel expression levels may be affected in the cells of ARPE19 by the deletion of TSPO. Hence, we tested TRPM2 channel expression levels in the cells of ARPE19, ARPE19-KO and SH-SY5Y. The expression level of TRPM2 in the SH-SY5Y cell was used as a positive control in the present study. We confirmed the presence of nature TRPM2 channel in the SH-SY5Y, ARPE19 and ARPE19-KO (Figure 1a,b and Supplementary Figure S1). The expression concentration of TRPM2 was higher in the ARPE19 as compared to the SH-SY5Y (*p* ≤ 0.05). However, the expression level of TRPM2 was downregulated in the ARPE19-KO cells by the deletion of TSPO. The expression concentration of TRPM2 was lower in the ARPE19-KO than in the ARPE19 cells (*p* ≤ 0.05).

### 3.2. The TRPM2 Was Activated in the ARPE19 by the Stimulation of H_2_O_2_

The modulator action of TSPO gene deletion on the VDCC and chemical-gated Ca^2+^ channels in several cells was recently reported reviewed in ref. [17], although there is limited report on the upregulation of [Ca^2+^]_c_ via the oxidative stress (H_2_O_2_)-mediated TRPM2 activation in the ARPE19 [21,23]. Hence, we checked whether the involvement of TSPO gene deletion on the upregulation of H_2_O_2_-mediated TRPM2 activation and [Ca^2+^]_c_ changes in the ARPE19. 

**Figure 2:**
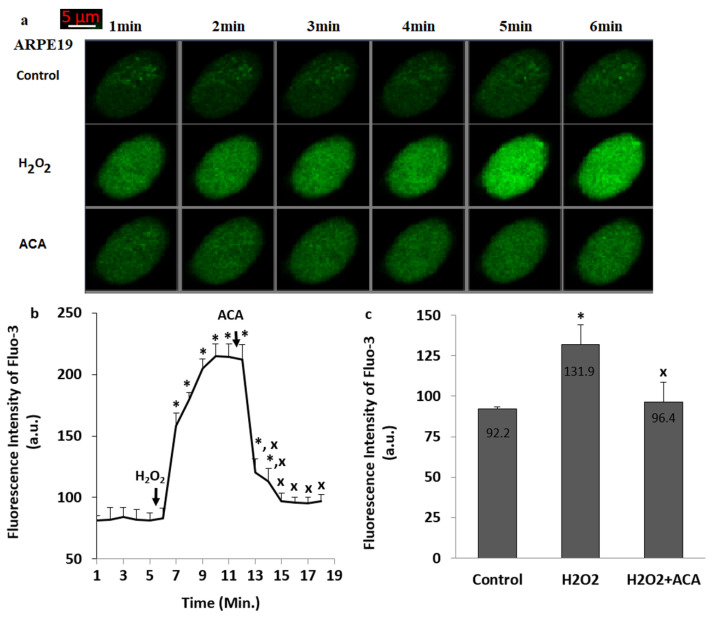
**The H_2_O_2_ -mediated upregulation of Ca^2+^ fluorescence intensity through the stimulation of TRPM2 in the ARPE19**. (n = 25–30). After incubating the ARPE19 cells with Fluo-3AM (1 µM for 60 min), the TRPM2 stimulator (H_2_O_2_ and 1 mM) and blocker (ACA and 25 µM for 6 min) applied to the cells within three groups (control, H_2_O_2_ and H_2_O_2_ + ACA). The cells were analyzed. The fluorescence intensity of Fluo-3AM in the ARPE19 was imaged at 515 nm in the LSM 800 laser scan microscope with 40 × 1.3 oil objective and the results were indicated as arbitrary unit (a.u.). (**a**) Imagines of the Fluo-3AM. (**b**,**c**) The mean fluorescence intensity of Ca^2+^ in the column and line figures were expressed as Mean ± SD in the ARPE19 after the treatments of H_2_O_2_ and ACA. (* *p* ≤ 0.05 vs. control. **^x^** *p* ≤ 0.05 vs. H_2_O_2_ group).

The green images of Ca^2+^ (Fluo-3AM) in the groups of control, H_2_O_2_ and H_2_O_2_+ACA of the ARPE19 were indicated in the Figure 2a. The [Ca^2+^]_c_ in H_2_O_2_ group was upregulated in the ARPE19 (Figure 2b,c) by the stimulation of H_2_O_2_ (1 mM) (*p* ≤ 0.05). However, the [Ca^2+^]_c_ was downregulated in the groups of H_2_O_2_ + ACA by the treatment of ACA (Figure 2b,c) (*p* ≤ 0.05). Hence, the H_2_O_2_ stimulation-mediated TRPM2 activation was observed in the ARPE19. 

### 3.3. The H_2_O_2_-Mediated Upregulation of [Ca^2+^]_c_ in the ARPE19 Was Diminished by the Treatments of PARP-1 Inhibitors (PJ34 and DPQ), TRPM2 Blocker (2APB) and GSH

**Figure 3:**
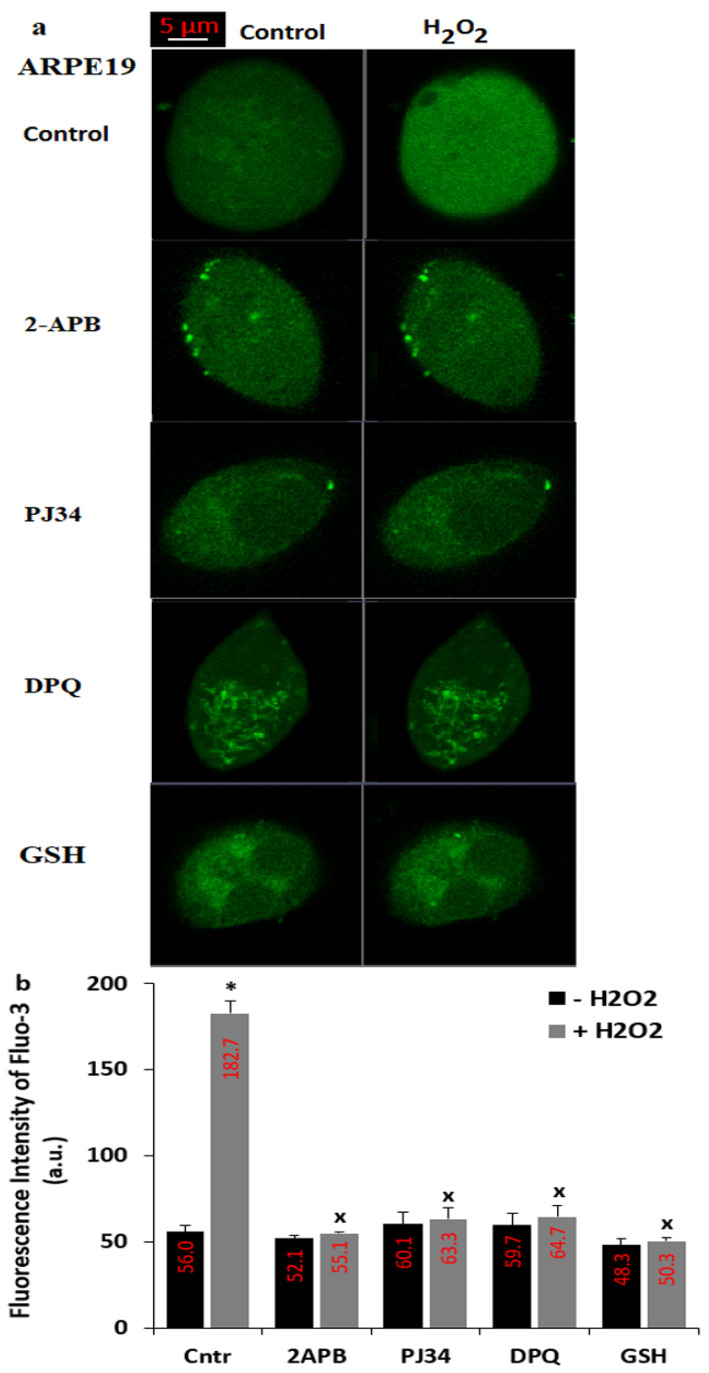
**There is no H_2_O_2_-mediated florescence change via TRPM2 activation in the presence of GSH, PARP-1, and TRPM2 blockers.** (Mean ± SD). The ARPE19 cells in the dishes were incubated with Fluo-3AM (1 µM for 60 min.). After pre-incubation of cells with the inhibitors of PARP-1 (1 µM PJ34 and 30 µM DPQ), TRPM2 (100 µM 2APB for 30 min) and GSH (10 mM for 2 h), the cells were stimulated by H_2_O_2_ (1 mM for 6 min). The symbolic images (**a**) and the mean intensity values (**b**) of the [Ca^2+^]_c_ from the groups of control (Cntr), 2APB, PJ34, DPQ and GSH groups were obtained. The images were captured in the LSM 800 laser confocal microscope with 40 × 1.3 oil objective and the results of Fluo-3AM florescence intensity were indicated as arbitrary unit (a.u.). The scale bar: 5 µm. One symbolic image from each group was selected from 25–30 cells of 6 independent investigations. (* *p* ≤ 0.05 vs. Cntr (− H_2_O_2_) group. ^x^ *p* ≤ 0.05 vs. vs. Cntr (+ H_2_O_2_) group).

**Figure 4:**
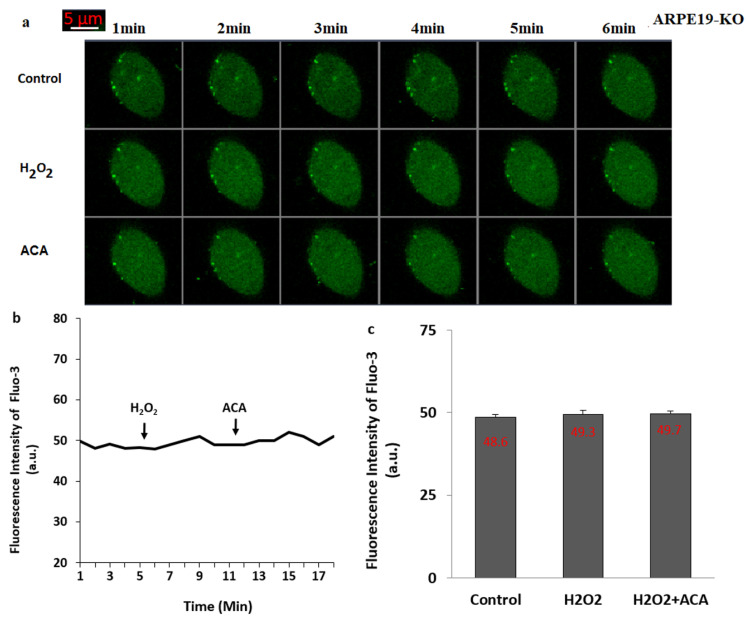
**The stimulation of H_2_O_2_ did not induce changes on the [Ca^2+^]_c_ and TRPM2 activation in the cells of ARPE19-KO**. (Mean ± SD). After incubating the cells of ARPE19-KO with Fluo-3AM (1 µM for 60 min), the TRPM2 stimulator (H_2_O_2_ and 1 mM) and blocker (ACA and 25 µM for 6 min) applied to the cells within three groups (control, H_2_O_2_ and H_2_O_2_ + ACA). The images of ARPE19-KO were captured in the LSM 800 with 40 × 1.3 oil objective. The scale bar was kept as 5 µm. The captured representative images (Figure 4a) and the mean Fluo-3AM intensity values (Figure 4b,c) in the groups of control (Cntr), H_2_O_2_ and H_2_O_2_ + ACA are shown in the Figure 4. One symbolic image from each group was selected from 25–30 cells of 6 independent investigations.

The current data indicate that the DNA-injury induced ADPR is produced in the nucleus of cells by the activation of PARP-1 enzyme [9]. The presence of ADPR pyrophosphatase enzyme in the C terminal NUDT9 homology domain of the TRPM2 was reported in the kidney cells (HEK293) [35,36] and ARPE19 cells [21,23]. The ADPR is intracellularly produced in the nucleus of cells by the enzymatic activity of PARP-1 [9]. However, the production of ADPR is reduced by the down-regulation of PARP-1 enzyme. The PJ34 and DPQ are well-known PARP-1 inhibitors [37] and their treatment induced the TRPM2 modulator action in the ARPE19 [21,23]. Importance of thiol redox system members such as glutathione (GSH) and selenium were reported on the TRPM2 activation in several cells, including ARPE19 [18,21,23], although effects of GSH on the TSPO-mediated TRPM2 activation in the ARPE19 cells has not been reported yet. Hence, we tested the protective actions of PJ34, DPQ, 2APB and GSH on the H_2_O_2_-mediated upregulations of TRPM2 and Ca^2+^ fluorescence intensity in the ARPE19 cells. The applications of extracellular H_2_O_2_ to the ARPE19 induced the upregulation of Ca^2+^ influx (the fluorescence intensity of Fluo-3AM) (Figure 3a,b) (*p* ≤ 0.05). However, the H_2_O_2_-mediated increase of Ca^2+^ influx was not observed in the cells by the preincubations of PJ34, DPQ, 2APB and GSH. The applications of extracellular H_2_O_2_ to the ARPE19-KO did not induce the upregulation of Ca^2+^ influx (the fluorescence intensity of Fluo-3AM) (Figure 4a–c), suggesting the involvement of TRPM2 in H_2_O_2_-induced Ca^2+^ influx in the ARPE19 cells.

### 3.4. The Treatment of ADPR Induced TRPM2 Activation in the ARPE19, But Not in the ARPE19-KO 

**Figure 5:**
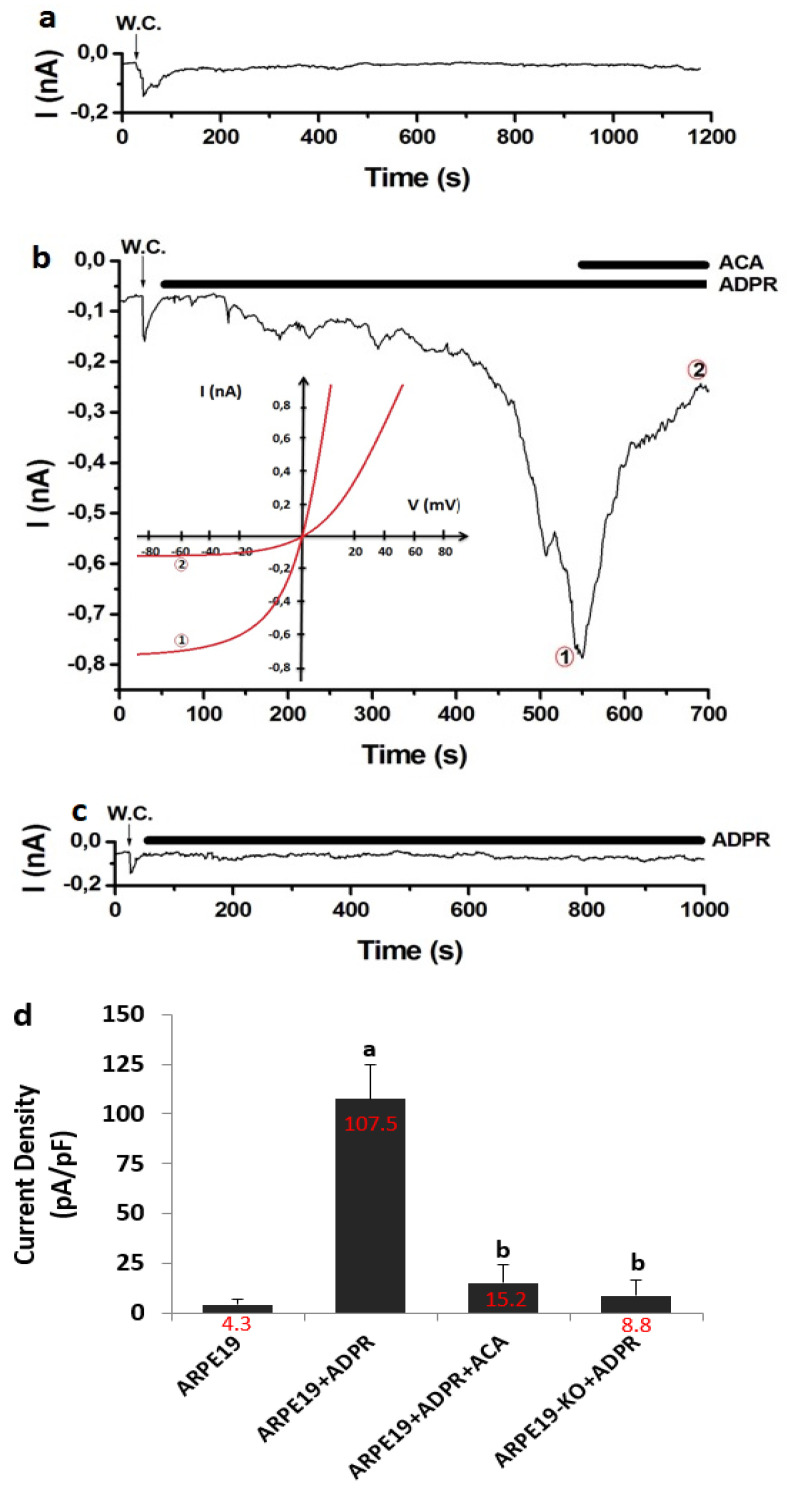
**The stimulation of ADPR induced the activation of TRPM2 in the cells of ARPE19, but not in the cells of ARPE19-KO.** (Mean ± SD and n = 6). The whole cell (W.C.) configuration current records of TRPM2 were taken in voltage-clamp (at −60 mV) (**a**) The cells of ARPE19 without cytosolic ADPR (1 mM). (**b**) ARPE19 + ADPR group. The cytosolic ADPR (1 mM)-mediated TRPM2 currents were inhibited by ACA (25 µM). (**b**)-I/V. Time points ADPR and ACA were indicated 1 and 2, respectively. (**c**) ARPE19-KO + ADPR group. There is no cytosolic ADPR (1 mM)-mediated TRPM2 current. (**d**) The mean current densities from the cells of ARPE19 and ARPE19-KO. (**^a^** *p* ≤ 0.05 vs. ARPE19). **^b^** *p* ≤ 0.05 vs. ARPE19 + ADPR).

TRPM2 was not gated in the absence of cytosolic ADPR, and there was no activation current of TRPM2 in the absence of ADPR in the patch pipette (Figure 5a). In the presence of ADPR in the patch-pipette, the increase of TRPM2 activation current was observed up to 0.78 nA in the ARPE19 + ADPR group. However, the increase of TRPM2 current was diminished to the control levels by the treatment of TRPM2 antagonist (ACA) (Figure 5b). The densities of TRPM2 currents were significantly (*p* ≤ 0.05) higher in the groups of ARPE19 + ADPR (107.52 pA/pF) than in the ARPE19 (4.31 pA/pF), although they were lower in the ARPE19 + ADPR + ACA group (15.16 pA/pF) than in the ARPE19 + ADPR group (107.52 pA/pF) (Figure 5d). In the ARPE19-KO cells (Figure 5c), the TRPM2 was not activated by the stimulation of cytosolic ADPR. The current densities of TRPM2 were markedly lower in the ARPE19-KO (8.84 pA/pF) as compared to the ARPE19 + ADPR group (107.52 pA/pF) (*p* ≤ 0.05) (Figure 3f). The cytosolic ADPR is specific agonist of TRPM2 and the absence of currents in the ARPE19-KO obviously evidenced the involvement of TPSO on TRPM2 activation in the ARPE19. 

### 3.5. The Levels of Apoptosis, Casp -3, Casp -9 and Cell Viability Were Affected in the ARPE19 But Not in the ARPE19-KO Cells by the H_2_O_2_ Stimulation: Modulator Role of ACA

Inactive forms of Casp -3 and Casp -9 locate in cytoplasm of cells, including ARPE19 [38,39]. In the pathways of apoptosis, Casp -3 and Casp -9 proteins have key roles. Overload Ca^2+^ influx via activation of many TRP channel subtype, including TRPM2 causes the upregulation of mMDP in several cells such as ARPE19 and SH-SY5Y [21,23]. In turn, the upregulation of mMDP induces apoptosis via the activations of Casp -3 and Casp -9. However, the ACA treatment-mediated TRPM2 inhibition caused to downregulation of Casp -3, Casp -9 and apoptosis, in many cells, including ARPE19 [19,21,23,28]. After finding the upregulation of [Ca^2+^]_c_ and TRPM2 currents in the ARPE19, we predicted the changes of apoptosis, Casp -3, Casp -9 and cell viability in the ARPE19. 

**Figure 6:**
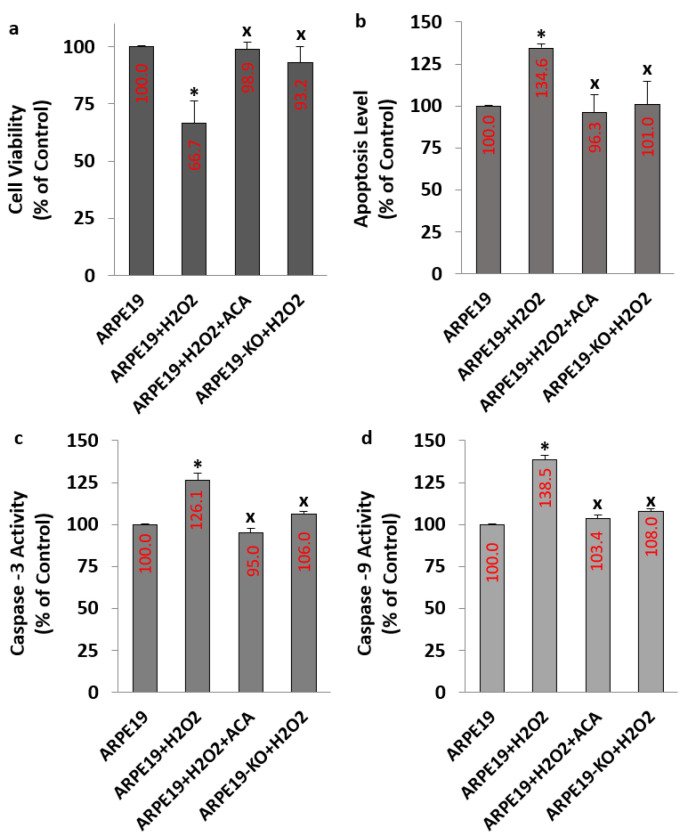
**The values of apoptosis, caspase -3 (Casp -3), and -9 (Casp -9) were upregulated, although the values of cell viability were downregulated in the cells of ARPE19 (but not in the cells of ARPE19-KO) by the stimulation of H_2_O_2_.** (Mean ± SD and n = 6). For analyzing cell viability, the test of MTT was used (**a**), whereas the value of apoptosis (**b**) was analyzed by using the ApoPercantage kit. The fluorogenic substrates of Ac-DEVD-AMC and *Ac*-LEHD-AFC were used for the assays of Casp -3 (**c**) and Casp -9 (**d**), respectively. (* *p* ≤ 0.05 vs. ARPE19. ^x^ *p* ≤ 0.05 vs. ARPE19 + H_2_O_2_).

The levels of apoptosis (Figure 6b), Casp -3 (Figure 6c) and Casp -9 (Figure 6d) were upregulated in the groups of ARPE19+H_2_O_2_, although the levels of cell viability were downregulated in the groups of ARPE19+H_2_O_2_ by the stimulation of H_2_O_2_ (*p* ≤ 0.05) (Figure 6a). However, the changes of Casp -3, Casp -9, cell viability and apoptosis in the groups of ARPE19 + H_2_O_2_ + ACA were modulated by the incubation of ACA (*p* ≤ 0.05). However, the changes were not observed in the ARPE19-KO by the stimulation of H_2_O_2_ or the inhibition of ACA (*p* ≥ 0.05). 

### 3.6. H_2_O_2_-Induced Increase of mMDP and Mito-fROS Levels Were Diminished in the ARPE19 Cells by the Treatment of 2APB

The involvement of TRPM2-mediated fROS generation on apoptosis in the several cells except ARPE19 was recently reported [19,23,25]. After observing the increase of apoptosis levels in the ARPE19, we predicted the increase of mMDP and Mito-fROS via the upregulation of TRPM2. 

**Figure 7:**
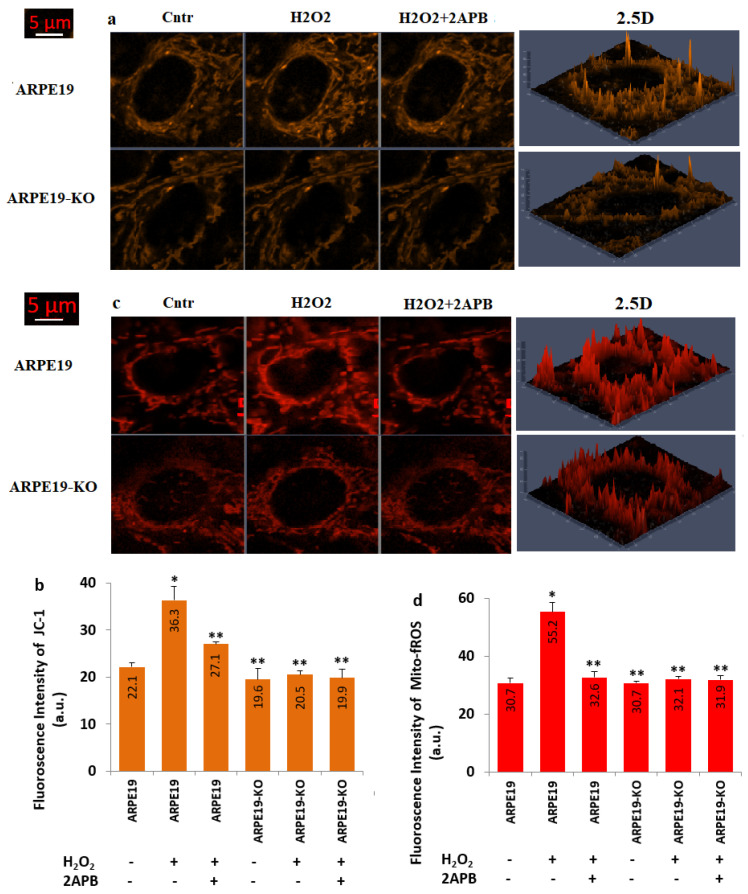
**The levels of Mito-Dep (JC-1) and mitochondrial fROS (Mito-fROS) were upregulated in the ARPE19, but not in the ARPE19-KO by the stimulation of H_2_O_2_.** (N = 25–30 and mean ± SD). After staining the cells with JC-1 (5 µM) and MitoTracker Red CM-H2ros (1 µM) dyes, the images of JC-1 and Mito-fROS were captured in the LSM 800 with the 40 × 1.3 oil objective. (**a**) The images of JC-1. (**b**) The mean fluorescence intensities of the JC-1 as arbitrary unit (a.u). (**c**) The images of MitoTracker Red CM-H2ros. (**d**) The mean fluorescence intensities of the Mito-fROS. (* *p* ≤ 0.05 vs. ARPE19 group. ** *p* ≤ 0.05 vs. ARPE19 + H_2_O_2_ group).

The levels of JC-1 (Figure 7a,b) and Mito-fROS (Figure 7c,d) were upregulated in the groups of ARPE19 + H_2_O_2_ as compared to the groups of ARPE19 (*p* ≤ 0.05). However, the treatments of 2APB downregulated the action of H_2_O_2_ on the JC-1 and Mito-fROS in the ARPE19 + H_2_O_2_ + 2APB group (*p* ≤ 0.05). The changes of the JC-1 and Mito-fROS values in the ARPE19-KO were not observed after the H_2_O_2_ and 2APB treatments. Hence, there were no changes on the values in the ARPE19-KO (*p* ≥ 0.05). The values of JC-1 and Mito-fROS were higher in the ARPE19 + H_2_O_2_ as compared to ARPE19-KO + H_2_O_2_. Therefore, the results of JC-1 and Mito-fROS further confirmed the action of TSPO on [Ca^2+^]_c_ and Mito-fROS-mediated apoptosis in the ARPE19.

### 3.7. The Levels of rGSH, GSHPx and MDA Were Modulated in the ARPE19 by the Deletion of TSPO 

**Figure 8:**
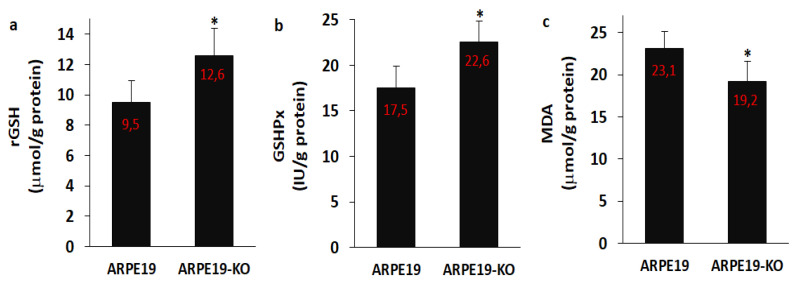
**The levels of reduced glutathione (rGSH), glutathione peroxidase (GSHPx) and lipid peroxidation (MDA) were modulated in the ARPE19-KO cells by the deletion of TSPO** (N = 6 and mean ± SD). The levels of rGSH (**a**), GSHPx (**b**) and MDA (**c**) were spectrophotometrically analyzed in the cells. (* *p* ≤ 0.05 vs. ARPE19).

After observing the increase of oxidative stress values, we suspected the decrease of antioxidant levels, namely rGSH and GSHPx. Hence, we analyzed the rGSH (Figure 8a), GSHPx (Figure 8b) and MDA (Figure 8c) levels in the ARPE19 and ARPE19-KO cells. The levels of rGSH and GSHPx were lower in the ARPE19 cells as compared to the ARPE19-KO cells, although the level of MDA was higher in the ARPE19 cells than in the ARPE-KO cells (*p* ≤ 0.05). 

### 3.8. The Expression Levels of PARP-1, Bax, Bcl-2, Casp -3, and Casp -9 Were Modulated in the ARPE19 by the Deletion of TSPO 

**Figure 9:**
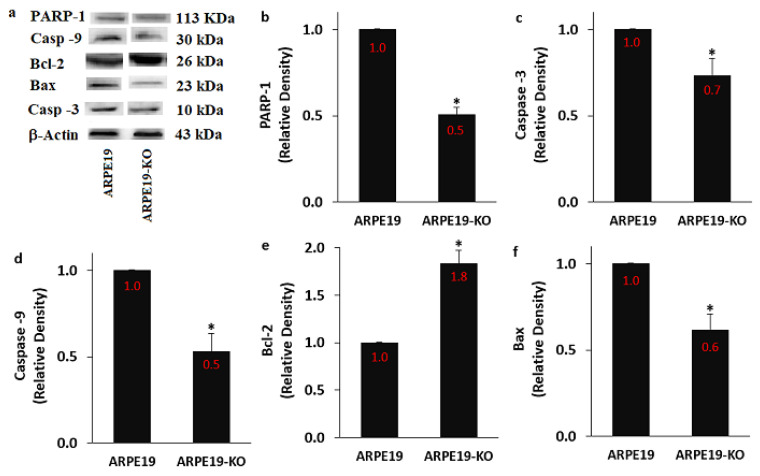
**The regulator role of TSPO deletion on the protein expressions of PARP-1, caspase-3 (Casp -3), caspase -9 (Casp -9), Bcl-2, and Bax.** (Mean ± SD and n = 3). The band expressions of PARP-1, Casp -3, Casp -9, Bcl-2 and Bax were determined by standard method of Western blot. The proteins of β-actin were used as control. (**a**) Western blot bands. The mean values of PARP-1 (**b**), Casp -3 (**c**), Casp -9 (**d**), Bcl-2 (**e**) and Bax (**f**) were indicated in the Figure 8 by columns. (* *p* ≤ 0.05 vs. ARPE19).

An interaction between the increase of TRPM2 expression and [Ca^2+^]_c_ was reported in ARPE19 [21,23]. It was reported that the generation of ADPR in cells was induced by the upregulation of PARP-1 activation [9]. In turn, ADPR-mediated stimulation of TRPM2 causes the upregulation of Bax, Casp -3 and Casp -9 expression levels, although its activation decreases Bcl-2 expression level in the ARPE19 [21,23]. However, data are not available on the expression values of Bax, Bcl-2, Casp -3, Casp -9 and PARP-1 in the ARPE19 after the deletion of TSPO gene. In the current data, the protein expression values of PARP-1 (Figure 9a,b and Supplementary Figure S2), Casp -3 (Figure 9c), Casp -9 (Figure 9d) and Bax (Figure 9e) were higher in the group of ARPE19 in comparison with the groups of ARPE19-KO (*p* ≤ 0.05). However, the protein expression level of Bcl-2 was upregulated in the ARPE19-KO group by the deletion of TSPO (*p* ≤ 0.05) (Figure 9f). 

## 4. Discussion

A mitochondria protein of the cellular outer membrane in the mitochondria of several cells, including ARPE19 is TSPO. Accumulating evince indicates that the presence of TSPO participated the modulations of Ca^2+^ homeostasis and mitochondrial fROS generation [10,11]. The deletion of TSPO gene provides to study the action of TSPO on the levels of apoptosis, ADPR, Mito-fROS and apoptosis via the stimulation of Ca^2+^ permeable channels in the models of cell culture [40,41,42,43]. The stimulations of oxidative stress and ADPR induce the activation of TRPM2 in the ARPE19 [21,23]. For clarifying the involvement of TSPO in retinal human diseases, we used the ARPE19 cell culture model [13]. The TSPO knockout ARPE19 lines were generated by means of CRISP TSPO-gRNA [33]. The current results demonstrated that the deletion of TSPO induces the regulation of TRPM2 in the ARPE19-KO, and it may present via the activations of anti-apoptotic and antioxidant pathways to inhibit the oxidant and apoptotic adverse actions of TSPO activation. In fact, the present results show that the presence of TSPO increased the upregulations of apoptosis and mitochondria oxidative cytotoxicity values via stimulation of TRPM2 in the ARPE19. Nevertheless, the blockages of PARP-1 (PJ34 and DPQ) and TRPM2 (2APB and ACA) downregulated the values of cell death and oxidative cytotoxicity in the ARPE19. Correspondingly, the decreases of cell death and oxidative cytotoxicity in the ARPE19-KO were decreased by the deletion of TSPO gene. In summary, present results clearly demonstrate that the deletion of TSPO decreases mitochondrial oxidative cytotoxicity-mediated cell death via the modulation of TRPM2 in the ARPE19. 

Present literature data indicated that the existence of TSPO induces the upregulation of mitochondrial redox signaling and PARP-1 activation, resulting in upregulated values of intracellular [Ca^2+^]_c_, causing to stimulation of the Ca^2+^-mediated NADPH oxidase, thereby upregulating fROS values [24,25]. The TRPM2 channel is stimulated by NADPH oxidase-induced fROS and PARP-1 activation-induced ADPR generations [22,35,36], although the channel is inhibited in several cells by the inhibitors of NADPH oxidase (apocynin), PARP-1 (DPQ and PJ34) and stimulation of GSH [21,22,31,37]. Similarly, the regulator role of TSPO deletion on the NADPH oxidase and PARP-1 activation in mice microglia was reported [24,25]. The modulator role of antioxidant GSH was reported on the TRPM2 activation in the ARPE19 [18,21,23]. In the present results, we remarked oxidative stress and ADPR-dependent TRPM2 activation in the ARPE19, while the TRPM2 stimulation was regulated by the GSH, PARP-1 blocker and TRPM2 antagonists. In addition, the stimulation was not remarked in the TSPO gene deleted cells (ARPE19-KO). The results clearly presented the interaction between the presence of TSPO and the TRPM2 stimulation in the ARPE19. 

The increases of apoptosis and cell death are induced by the excessive Ca^2+^ accumulation into mitochondria [44]. Even though the role of excessive Ca^2+^ influx-mediated programmed cell death in neuronal injury has been discussed in several studies [21,23,30,31], TRPM2 activation-mediated apoptosis has not been explained in the ARPE19 yet. In the presence of excessive Ca^2+^ influx into cytosol of retinal pigment epithelium, the pore of mitochondrial permeability transition (mPTP) has been shown to open in response to the mitochondrial Ca^2+^ overload and ATP depletion [45]. Opening the mPTP via TRPM2-induced overload Ca^2+^ influx leads to the increase of mMDP. In turn, it induces the increases of Bax, programmed cell death, Casp -3 and Casp -9 in the ARPE19, although it induces the decrease of cell viability and Bcl-2 in the cells [21,23]. It was reported that PARP-1 activation causes oxidative damage via opening the mPTP in the mitochondria [46,47], although its inhibition via PJ34 and DPQ regulates mitochondrial oxidative damage [9,18,19]. One of the MPTP components is TSPO. The opening of the mPTP is arranged by the TSPO gene [48,49]. TRPM2 activator, ADPR, is produced in the nucleus of several cells by the stimulation of PARP-1 [9]. TRPM2 is regulated by the inhibition of PARP-1 [37]. Therefore, we hypothesized that the inhibitions of PARP-1 and TRPM2 regulate the TSPO-induced upregulation of apoptosis, Bax, Casp -3 and Casp -9 in the TSPO deleted ARPE19. In this study, the decreases of Bax, programmed cell death, Casp -3 and Casp -9 were found in ARPE19 after TSPO deletion. Moreover, the deletion of TSPO gene and the treatments of TRPM2 antagonists prevented the oxidative stress-induced programmed cell death, Casp -3 and Casp -9 in ARPE19. Thus, the effects of TSPO on oxidative stress-induced programmed cell death, Casp -3 and Casp -9 were dependent of the activations of PARP-1 and TRPM2 in the ARPE19. 

The rate of oxygen consumption is high in the retina [1]. A large amount of fROS generation in the ARPE19 cells from a variety of cellular sources, including mitochondria [1]. The activation of TRPM2 induces overload Ca^2+^ influx-mediated oxidative stress by the gating mPTP and injuring mMDP [9,18,19]. The influxes of Ca^2+^ also action as a direct stimulus to cause TRPM2 stimulation-induced fROS in the ARPE19 [21,23]. The deletions of TSPO regulate mPTP activity via the inhibition of VDCC activation by decreasing the mitochondrial fROS generation, Ca^2+^ influx and mitochondrial Ca^2+^ accumulation in several cells [14,40]. PARP-1 can be overstimulated by fROS and PARP-1. In turn, it also stimulates the activations of TRPM2 and oxidant redox system [9]. In accordance with the PARP-1 and fROS reports, we observed that the inhibition of TRPM2 was able to downregulate the generations of fROS after TSPO deletion. Moreover, the upregulation of mitochondrial fROS was downregulated when the cells were pre-treated with ACA and 2APB. Thus, fROS is possibly a downregulation signal, which TRPM2 modulates mMDP. The present results confirm recent results, which show that the TSPO gene deletions in the microglia of human and mouse led to the downregulation of mMDP, intracellular Ca^2+^ homeostasis and mitochondrial respiratory homeostasis [12]. The activations of phagocytic cells and microglia cause to the excessive generation of fROS for killing bacteria and viruses, although the excessive generation of fROS induces adverse action in the normal tissues, including retina [1,8]. Klee et al. [50] investigated the involvement of TSPO gene deletion on the microglia and macrophage activation-induced retinal degeneration in mice and human. Contrary to the present results, they were not able to found the relationship between the deletion of TSPO and the changes of retina in mice and human. 

The fROS is scavenged by several antioxidants. The GSHPx as an antioxidant enzyme scavenges the hydrogen peroxide and hydroxyl radicals by using GSH as substrate [9,21]. The increase of fROS and oxidative stress causes the decrease of antioxidant levels, namely rGSH and GSHPx in the ARPE19 cells, although the treatment of GSH increases antioxidant levels via the decrease of MDA level and TRPM2 activity in several cells [18,21,23]. In the current data, the levels of rGSH and GSHPx were increased in the ARPE19-KO cells by the deletion of TSPO, although the level of MDA was decreased in the cells. The results of rGSH, GSHPx and MDA confirmed presence of a relationship between the GSH redox system and TSPO-mediated oxidative stress in the ARPE19 cells. 

## 5. Conclusions

In summary, the deletion of TSPO protects ARPE19 from mitochondrial oxidative stress-mediated programmed cell death via the block of TRPM2-mediated fROS generation in the mitochondria and to the inhibitions of Casp -3 and Casp -9. The action of TSPO on the oxidant and programmed cell death pathways in the ARPE19 were performed by the upregulation of the molecular TRPM2-mediated excessive Ca^2+^ influx pathways (Graphical Abstract). According to the present data, we concluded that the deletion of TSPO caused the inhibition of TRPM2. Hence, inhibiting the TRPM2 may defend the ARPE19 against mitochondrial oxidative injury and programmed cell death. Therefore, the deletion of TSPO may be considered as a beneficial strategy against oxidative injury-mediated ARPE19 cell death.

## Data Availability

Present cell culture methods in the present study were performed in the BSN Health, Analyses, Innovation, Consultancy, Organization, Agriculture and Industry Ltd. (Isparta, Turkey). The details of present dataset and analyses are available from the Mustafa Nazıroğlu on reasonable request.

## Supplementary Materials

The following are available online at https://www.mdpi.com/article/10.3390/biology10050382/s1, Figure S1: The row data of TRPM2 protein expression level in the SH-SY5Y, ARPE19, and ARPLE19-KO cells, Figure S2: The row data of PARP-1, caspase-3 (Casp -3), caspase -9 (Casp -9), Bcl-2, and Bax in the ARPE19 and ARPE19-KO cells.

## Abbreviations

[Ca^2+^]_c_	concentration of intracellular free calcium ion
2APB	2-aminoethoxydiphenyl borate
ACA	N-(p-amylcinnamoyl)anthranilic acid
ADPR	ADP-ribose
AMD	age-related macular degeneration
ARPE19	adult retinal pigment epithelial 19
Casp -3	caspase -3
Casp -9	caspase -9
Ca^2+^	calcium ion
CRISPR	clustered regularly interspaced short palindromic repeats
fROS	free reactive oxygen species
GSH	glutathione
GSHPx	glutathione peroxidase
h-RPE	human retinal pigment epithelium
MDA	malondialdehyde
mMDP	mitochondrial membrane depolarization
mPTP	* mitochondrial * permeability transition pore
rGSH	reduced glutathione
TRP	transient receptor potential
TRPM2	transient receptor potential melastatin 2
TSPO	18 kDa mitochondrial translocator protein
VDCC	anion protein of voltage-dependent channel
