# Supplementary material for: Deletion of Mitochondrial Translocator Protein (TSPO) Gene Decreases Oxidative Retinal Pigment Epithelial Cell Death via Modulation of TRPM2 Channel
Source: Biology (Basel). 2021 Apr 28;10(5):382. doi: 10.3390/biology10050382 (PMC8145237; doi:10.3390/biology10050382)
Supplement: Supplementary file 1 [file biology-10-00382-s001.zip › biology-1127222-supplementary.pdf]

Deletion of mitochondrial translocator protein (TSPO) gene decreases oxidative retinal pigment epithelial cell death via modulation of TRPM2 channel

Dilek Özkaya[1], Xinhua Shu[2,3,4], Mustafa Nazıroğlu[5,6]

[1]Department of Ophthalmology, Faculty of Medicine, Suleyman Demirel University, Isparta, Turkey

[2]Department of Biological and Biomedical Sciences, Glasgow Caledonian University, Glasgow G4 0BA, UK

[3]Department of Vision Science, Glasgow Caledonian University, Glasgow G4 0BA, UK

[4]School of Basic Medical Sciences, Shaoyang University, Shaoyang, P.R. China

[5]Neuroscience Research Center, Suleyman Demirel University, Isparta, Turkey

[6]Drug Discovery Unit, BSN Health, Analyses, Innovation, Consultancy, Organization, Agriculture, Ltd., Isparta, Turkey

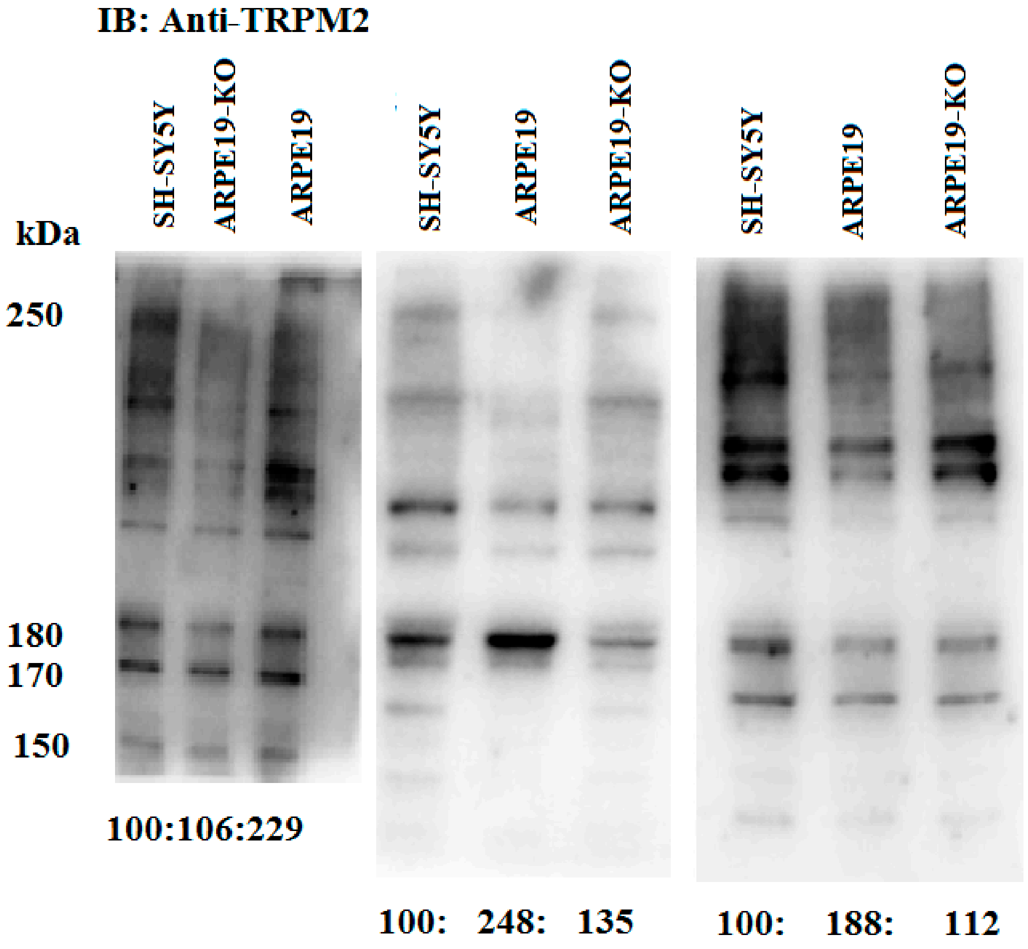

Supplementary Figure 1. The row data of TRPM2 protein expression level in the SH-SY5Y, ARPE19, and ARPE19-KO cells. (Mean ± SD and n=3). For the expression levels of TRPM2 protein in the cells of SH-SY5Y, ARPE19, and ARPE19-KO, we used standard Western blot analyses. The protein bands of β-actin were used as control. 1:500. (b) Densitometry readings/intensity ratio, using ImageJ software and were normalized to the corresponding β-Actin value. The results were expressed in relative density changes.

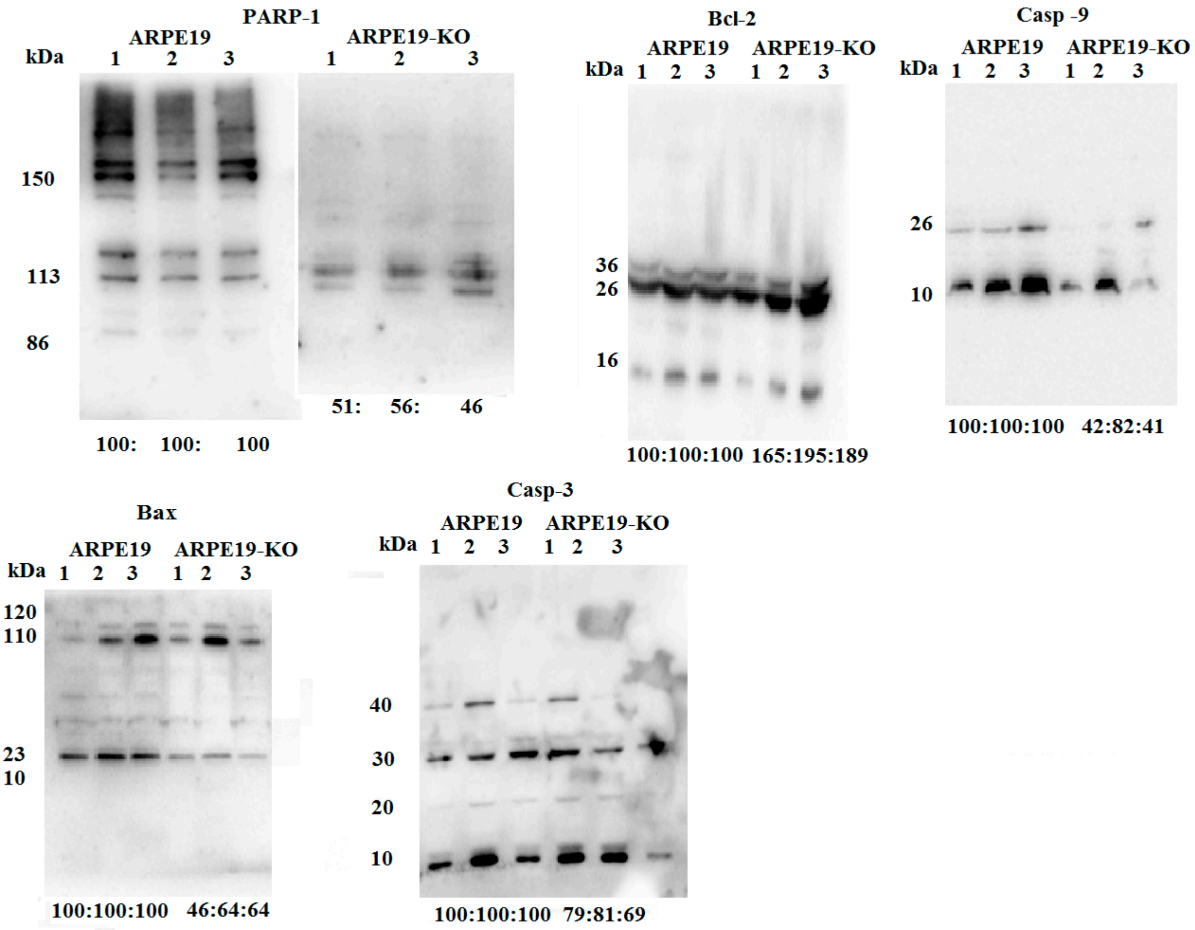

Supplementary Figure 2. The row data of PARP-1, caspase-3 (Casp -3), caspase -9 (Casp -9), Bcl-2, and Bax in the ARPE19 and ARPE19-KO cells. (Mean ± SD and n=3). For the protein expression levels in the cells of ARPE19 and ARPE19-KO, we used standard Western blot analyses. The protein bands of β-actin were used as control. The protein bands of β-actin were used as control. 1:200-1:1000. (b) Densitometry readings/intensity ratio, using ImageJ software and were normalized to the corresponding β-Actin value. The results were expressed in relative density changes.
